# Clinical Integration of Digital Patient-Reported Outcome Measures in Primary Health Care for Chronic Disease Management: Protocol for a Systematic Review

**DOI:** 10.2196/48155

**Published:** 2023-08-18

**Authors:** Maxime Sasseville, Wilfried Supper, Jean-Baptiste Gartner, Géraldine Layani, Samira Amil, Peter Sheffield, Marie-Pierre Gagnon, Catherine Hudon, Sylvie Lambert, Eugène Attisso, Victoria Bureau Lagarde, Mylaine Breton, Marie-Eve Poitras, Pierre Pluye, Pierre-Henri Roux-Levy, James Plaisimond, Frédéric Bergeron, Rachelle Ashcroft, Sabrina Wong, Antoine Groulx, Nicolas Beaudet, Jean-Sébastien Paquette, Natasha D'Anjou, Sylviane Langlois, Annie LeBlanc

**Affiliations:** 1 Université Laval Québec, QC Canada; 2 VITAM Research Center Québec, QC Canada; 3 Université de Montréal Montréal, QC Canada; 4 University of Toronto Toronto, ON Canada; 5 Université de Sherbrooke Sherbrooke, QC Canada; 6 Université McGill Montréal, QC Canada; 7 St. Mary's Research Center Montréal, QC Canada; 8 Division of Intramural Research National Institute of Nursing Research National Institute of Health Bethesda, WA United States; 9 Omnimed Sherbrooke, QC Canada

**Keywords:** systematic review, patient-reported outcome measure, primary healthcare, health care, implementation science

## Abstract

**Background:**

Health measurement guides policies and health care decisions are necessary to describe and attain the quintuple aim of improving patient experience, population health, care team well-being, health care costs, and equity. In the primary care setting, patient-reported outcome measurement allows outcome comparisons within and across settings and helps improve the clinical management of patients. However, these digital patient-reported outcome measures (PROMs) are still not adapted to the clinical context of primary health care, which is an indication of the complexity of integrating these tools in this context. We must then gather evidence of their impact on chronic disease management in primary health care and understand the characteristics of effective implementation.

**Objective:**

We will conduct a systematic review to identify and assess the impact of electronic PROMs (ePROMs) implementation in primary health care for chronic disease management. Our specific objectives are to (1) determine the impact of ePROMs in primary health care for chronic disease management and (2) compare and contrast characteristics of effective ePROMs’ implementation strategies.

**Methods:**

We will conduct a systematic review of the literature in accordance with the guidelines of the Cochrane Methods Group and in compliance with the Preferred Reporting Items for Systematic Reviews and Meta-Analyses guidelines for its reporting. A specific search strategy was developed for relevant databases to identify studies. Two reviewers will independently apply the inclusion criteria using full texts and will extract the data. We will use a 2-phase sequential mixed methods synthesis design by conducting a qualitative synthesis first, and use its results to perform a quantitative synthesis.

**Results:**

This study was initiated in June 2022 by assembling the research team and the knowledge transfer committee. The preliminary search strategy will be developed and completed in September 2022. The main search strategy, data collection, study selection, and application of inclusion criteria were completed between October and December 2022.

**Conclusions:**

Results from this review will help support implementation efforts to accelerate innovations and digital adoption for primary health care and will be relevant for improving clinical management of chronic diseases and health care services and policies.

**Trial Registration:**

PROSPERO International Prospective Register of Systematic Reviews CRD42022333513; https://www.crd.york.ac.uk/prospero/display_record.php?RecordID=333513

**International Registered Report Identifier (IRRID):**

DERR1-10.2196/48155

## Introduction

Primary health care is essential, comprehensive, and universally available to a community [1]. For most of the Canadian population, primary health care represents the first contact with the health care system, and in 2016, overall, 88.5% of the Canadian population had access to a family physician attached to a primary health care service [2]. Mostly managed in primary health care, chronic diseases affect 3 out of 5 Canadian adults, significantly impacting their quality of life, and account for approximately 80% of disability and 70% of deaths in Canada [3]. Primary health care management of chronic diseases remains a challenge at the core of the health care system.

Health measurement guides policies and health care decisions and is necessary to describe and attain the quintuple aim of improving patient experience, population health, care team well-being, health care costs, and equity [4]. In the primary care setting, health measurement allows outcome comparisons within and across settings and helps improve the clinical management of patients [4,5]. However, for these outcomes to be informative, researchers, key stakeholders, clinicians, and patients must codevelop a measurement framework integrating the perspectives of end users [5,6] and for outcome measurement to deliver on the promise of addressing the quintuple aim [5].

Patient-reported outcome measures (PROMs) are health reports of patients’ conditions, capturing their perception of their own health, without clinician input [5]. PROMs enable patients to report on their quality of life, daily functioning, symptoms, and other aspects of their health and well-being [5]. A body of evidence shows that effective use of PROMs in the clinical context reduces consultation time [7], improves patient-provider communication [8], identifies patient needs and allows for a patient-centered practice [9], decreases symptom severity, increases survival, and decreases emergency department visits [10,11].

The development of integrated digital solutions to care pathways is essential for PROMs to have a clinical impact [8,12,13]. It has been reaffirmed in the 2021-2026 Canadian Institutes for Health Research’s strategic plan that researchers must accelerate digital transformation of health care systems for efficient health measurement and data analysis [4]. Electronic PROMs (ePROMs) can bring several benefits to the use of pen-and-paper PROMs: higher motivation to complete the task of self-reporting, lower rates of missing data and errors, improved chronology on historical patients' data [14], remote and quick data collection, integrated data analysis, and availability of reports for care teams [15].

However, ePROMs are still not adapted to the clinical context of primary health care, which is an indication of the complexity of integrating these tools in this context [16,17]. The effectiveness of including ePROMs in routine clinical care has been studied in contexts of specialized care among homogenous patient groups based on diagnoses such as cancer [18-24]. Whereas successful ePROMs implementation in Canada has been noted (eg, Cancer Care Alberta), unfortunately, there are additional implementation challenges specific to primary health care, including the heterogeneity of the patient population [25]. There are also significant disparities among patients regarding their ability to understand and use digital tools [26]. The development of ePROMs is expected to increase the existing digital divide and reduce the ability to self-report outcomes among certain populations [26]. Understanding the extent to which ePROMs provide outcomes for clinicians and patients and prioritize the adaptation of additional ePROMs in this context is critical to present exhaustive evidence of what works and how to implement ePROMs in primary health care. Consequently, to address these abovementioned critical needs and challenges, we must gather evidence of the impact of ePROMs on chronic disease management in primary health care and understand the characteristics of effective implementation.

With the goal of accelerating the integration of ePROMs to optimize the delivery of primary health care and bring about rapid and meaningful benefits to patients, we will conduct a systematic review to identify and assess the impact of ePROMs implementation in primary health care for chronic disease management.

Our objectives are to (1) determine the impact of ePROMs in primary health care for chronic disease management and (2) compare and contrast the characteristics of effective ePROMs implementation strategies. We expect that our results will directly contribute to providing decision makers with high-quality, timely, accessible, and relevant evidence to inform policies and practices.

## Methods

We will conduct a systematic review of the literature in accordance with the guidelines of the Cochrane Methods Group and in compliance with the PRISMA (Preferred Reporting Items for Systematic Reviews and Meta-Analyses) guidelines for its reporting [27,28]. We have registered the study protocol to the PROSPERO systematic review registry (CRD42022333513) [29].

### Conceptual Underpinnings

This review will be grounded in the integrated knowledge translation approach to pursue its objectives [30]. According to this approach, knowledge users (KUs) will be involved in all aspects of the research process to enhance its relevance and usability in practice. The Strategy for Patient-Oriented Research framework for patient engagement will be used to complete integrated knowledge translation in promoting a respectful climate between researchers and KUs, including decision makers and patient partners [31].

### Synthesis Questions

The following questions will be addressed: (1) what are the outcomes of ePROMs in primary health care in chronic disease management? (2) What are the effective strategies to implement ePROMs in primary health care? (3) What are the challenges, barriers, and facilitators to successful implementation of ePROMs in primary health care?

### Eligibility Criteria

We will address all types of evidence matching the Population, Intervention, Comparison, Outcomes, and Setting/context criteria [28]. *Participants* include all studies reporting on the implementation of an ePROM among adults for chronic disease management. We have no restrictions regarding the *Interventions/phenomena of interest*; we will include all types of implementations, theoretical models, structures or PROMs. We have no restrictions regarding the *Comparator*; we will consider all outcomes reported in the studies. We will seek outcomes related to patients, caregivers, health care providers, policy makers, barriers, facilitators, acceptability, feasibility, adoption, fidelity, morbidity, mortality, quality of life, satisfaction, cost and cost-effectiveness. Regarding *Setting*, we will only include studies taking place in primary health care settings in any geographical setting and extract information regarding implementation. All types of studies will be included (ie, those using qualitative, quantitative, and mixed methods), which were published in French, English, or Spanish.

### Search Strategy

We will develop the search strategy under the leadership and guidance of an experienced information specialist (FB). An iterative process of revision by the research team members and a revision by another experienced information specialist will be carried out (per the PRESS [Peer Review of Electronic Search Strategies] checklist) [32]. All relevant comments will be integrated in the final version of the search strategy. Specific search strategies will be formulated for the following databases: Cochrane Database of Systematic Reviews, MEDLINE (Ovid), CINAHL (EBSCO), Embase, PsycINFO (Ovid), Web of Science, and ProQuest Dissertations & Theses. Considering the recent nature of the topic, a time limit of 20 years will be applied to the search strategies. No restriction on language will be applied. We will develop and include a gray literature search strategy. We will perform an internet search in the following sources and search engines: Google, Google Scholar, Canadian Evaluation Society, EuroScan, OpenGrey, Grey Literature Report, GreyNet, and Grey Matters. Since innovation implementations are often conducted by government entities or large health care systems, we will search within the websites of key governmental agencies (eg, US Agency for Healthcare Research and Quality’s studies on health care systems [eg, Kaiser Permanente]). Furthermore, we will perform backward citation searches of included studies and recent literature reviews for additional relevant references.

### Data Collection and Screening

We will export all citations in the web-based collaboration tool Covidence, where duplicated entries will be removed with the automation function [33]. Titles and abstracts will be screened independently by pairs of reviewers. A pilot testing to validate the screening process will be completed on 5% of a random sample of all studies. Variability in reviewer assessment will be documented using the Cohen κ statistic [34]. Ambiguous or incomplete abstracts will be retained to be reviewed in full and additional information could be obtained from the authors. We will search and obtain all the full texts of the selected references and will import the PDF files in Covidence. Pairs of reviewers will independently apply the inclusion criteria using the full texts following a pilot testing using the process outlined above. At any moment in the overall screening process, a third reviewer will help resolve any discrepancy. All the reasons for exclusion will be recorded in Covidence. A PRISMA flowchart will be used to describe study identification, screening, inclusions, and exclusions [35].

### Data Extraction and Appraisal

We will codevelop the extraction form with all KUs and team members to ensure that we are capturing relevant information (ie, what matters the most for them). Pairs of reviewers will independently complete data extraction following a pilot testing (see above). We will extract descriptive data (title, year of publication, authors, funding, conflicts of interests, and country), study types (published or gray literature), methodological data (design, sample size, measure constructs, and name of the instrument), setting data (clinical setting, type health professionals, and patient population), implementation data (description of implementation activities, facilitators, and barriers), outcomes (patient health, providers workflow, and cost), and outcome types (qualitative and quantitative). The quality of the included studies will be evaluated using the mixed methods appraisal tool [36]—a tool adapted to systematic reviews synthesizing data from qualitative, quantitative, and mixed methods studies.

### Data Synthesis

We will use the RE-AIM (reach, effectiveness, adoption, implementation, maintenance/sustainability) framework as a data analysis framework. The RE-AIM framework has been developed to evaluate the public health impacts of interventions and has been used in systematic reviews to help structure the assessment of the different implementation factors at play in complex contexts and settings [37,38]. This framework includes 5 dimensions: reach (how willing the targeted population is to participate in the intervention; ie, ePROMs), efficacy (what is the impact of the intervention on outcomes?), adoption (can this be adopted by new groups with ease and minimal changes?), implementation (what are the special issues and barriers?), and maintenance (can the intervention be maintained and its impact sustained?). The use of the RE-AIM framework will enable us to provide an overview of the parameters strengthening (review questions 2 and 3) the efficiency (review question 1) of ePROMs’ integration in primary health care and its impact on outcomes associated with the quintuple aim.

We will use a 2-phase sequential mixed methods synthesis design (ie, conduct a qualitative synthesis) and use its results to perform a quantitative synthesis [39]. In phase 1 (qualitative), we will use a thematic synthesis procedure [40] wherein we will summarize and describe methods and approaches designed to implement and integrate ePROMs in primary care. The qualitative data synthesis will produce structured narrative summaries of main themes of data in accordance with the RE-AIM framework. In phase 2 (quantitative), for each phase 1 theme, we will synthesize quantitative data with study subgroups centered around the theme in a descriptive manner when statistics and calculation methods are available. We will summarize study characteristics and methodological differences and similarities to highlight the following points: strengths and weaknesses of each implementation method, main outcomes of implementation, main conclusions regarding the relationship between methods and outcomes, main resources used and their impacts, and whether any trade-offs are described and their effect on the results of the study. If data are available in the primary studies, sex and gender similarities and differences (eg, adoption and satisfaction) will be included in our synthesis. The diversity in team expertise, clinical background, and gender will contribute to the orientation of the synthesis and choices that could lead to inclusive recommendations for clinical choices and policies. This study will also follow the Canadian Institutes for Health Research’s SAGER (Sex and Gender Equity in Research) for study design and reporting of the results. If needed, we will also provide training in sex and gender equity for the trainees and the patient partners on the team [41].

## Results

The study was initiated in June 2022 by assembling the research team and the knowledge transfer committee. The preliminary search strategy was developed and completed in September 2022. The main search strategy, data collection, study selection, and application of inclusion criteria were completed between October and December 2022. Data analysis will be completed and reports, briefs, and papers will be written from January to September 2023. The knowledge translation committee meets throughout the study period, once every 2 months, but more actively, from January to March 2023.

## Discussion

In order to not only impact but also document the extent of success toward the abovementioned quintuple aim, health measurements must include patients’ self-evaluation of health, well-being, and behaviors that contribute to care quality, service comparability, provider performance, patient engagement, and general patient status. Effective implementation of relevant ePROMs in primary health care is necessary to orient chronic disease management that is patient-centered and personalized in a contemporary and modern context of care. This study aims to synthesize the learning from using ePROMs in primary health care organizations. This information will be useful in supporting the adoption of PROMs in clinical settings. Optimal implementation of PROMs in primary health care would improve perceived relevance and adoption by health care providers and patients. Results from this review will orient the delivery, accountability, effective implementation, and contextualized usefulness of PROMs for the implementation of ePROMs in clinical settings. Optimal implementation of ePROMs in primary health care would improve perceived relevance and adoption by health care providers and patients. Results from this review will orient the delivery, accountability, effective implementation, and contextualized usefulness of PROMs. The patient-centered approach facilitated by PROM use will greatly improve health equity for populations needing a personalized care approach of primary health care.

Results from this review will help support implementation efforts to accelerate innovations and digital adoption for primary health care and will be relevant for improving clinical management of chronic diseases, health care services, and policies. Efficient ePROM implementation has a direct impact on the quintuple aim of improving patient experience, improving health, improving health professionals experience, improving care value, and advancing health equity.

## Appendix

Multimedia Appendix 1 Peer-review report.

## Abbreviations

ePROM: electronic patient-reported outcome measure
KU: knowledge user
PRESS: Peer Review of Electronic Search Strategies
PRISMA: Preferred Reporting Items for Systematic Reviews and Meta-Analyses
PROM: patient-reported outcome measure
RE-AIM: reach, effectiveness, adoption, implementation, maintenance/sustainability
SAGER: Sex and Gender Equity in Research

## Data Availability

The data sets generated during and/or analyzed during this study are available from the corresponding author on reasonable request.
