# Supplementary material for: Clinical Integration of Digital Patient-Reported Outcome Measures in Primary Health Care for Chronic Disease Management: Protocol for a Systematic Review
Source: JMIR Res Protoc. 2023 Aug 18;12:e48155. doi: 10.2196/48155 (PMC10474503; doi:10.2196/48155)
Supplement: Multimedia Appendix 1 [file resprot_v12i1e48155_app1.pdf]

February/Février 28, 2022

DC0190GP

Canadian Institutes of Health Research / Instituts de recherche en santé du Canada

Notice of Decision / Avis de décision

Application Number/Numéro de la demande: 475355

Committee Code/Code du comité: QAE

Applicants/Candidats: Docteur Annie Leblanc, Docteur Antoine Groulx

With/Avec:

Dr. R. Ashcroft	Docteur N. Beaudet	Prof. M. Breton	Docteure M. Gagnon
Dr. C. Hudon	Dr. S. Lambert	Docteur J. Paquette	Docteur P. Pluye
Professeure M. Poitras	Docteur M. Sasseville	Dr. S. Wong	

Institution paid/ Université Laval

Title/Titre: Clinical integration of digital patient-reported outcomes in primary healthcare for chronic diseases management - a systematic review (inPR1nt)

Primary Inst./ Inst. principal: Health Services and Policy Research / Services et politiques de la santé

Other Related Inst./ Autres inst. connexes: Population and Public Health / Santé publique et des populations

Competition Outcome/Résultats du concours: Catalyst Grant: Quadruple Aim and Equity / Subvention Catalyseur : Quatre objectifs et équité

October/Octobre 26, 2021

Number in competition/Nbre de demandes dans le concours: 72

Number approved/Nbre de demandes approuvées: 50

Decision on your application/ Décision sur votre demande: Approved / Approuvée

Average annual amount/ Montant annuel moyen: $99,810

Term/Durée: 1 **yrs/ans** 0 **months/mois**

Peer Review Committee Recommendation, for your information and use/
Recommandation du comité d'examen par les pairs, pour fins d'information et d'utilisation:

Committee/Comité: Catalyst Grant : Quadruple Aim and Equity / Subvention catalyseur : Quatre objectifs et équité

Application rank within the competition/ 11

Percent Rank Within the Competition/ 15.28%

Rating/ Mérite scientifique 4.53

Répercussions possibles 4.05

Recommended average annual amount/ Montant annuel moyen recommandé: $99,810

*** Applications receiving a score of less than 3.5 on any evaluation criteria will not be considered for Funding. / Les demandes qui ont reçu une note inférieure à 3.5 pour n'importe quel des critères d'évaluation ne sont pas admissibles.

This document is for information only. Official payment is stated on the CIHR Authorization for Funding.
Document à titre d'information seulement. Le paiement officiel est indiqué sur l'autorisation de financement des IRSC.

Canadian Institutes of Health Research
160 Elgin Street, 9th Floor
Address Locator 4809A
Ottawa, Ontario K1A 0W9

Instituts de recherche en santé du Canada
160, rue Elgin, 9e étage
Indice de l'adresse 4809A
Ottawa (Ontario) K1A 0W9

Institute of Aging
Institute of Cancer Research
Institute of Circulatory and Respiratory Health
Institute of Gender and Health
Institute of Genetics
Institute of Health Services and Policy Research
Institute of Human Development and Child and Youth Health
Institute of Indigenous Peoples' Health
Institute of Infection and Immunity
Institute of Musculoskeletal Health and Arthritis
Institute of Neurosciences, Mental Health and Addiction
Institute of Nutrition, Metabolism and Diabetes
Institute of Population and Public Health

Institut du vieillissement
Institut du cancer
Institut de la santé circulatoire et respiratoire
Institut de la santé des femmes et des hommes
Institut de génétique
Institut des services et des politiques de la santé
Institut du développement et de la santé des enfants et des adolescents
Institut de la santé des Autochtones
Institut des maladies infectieuses et immunitaires
Institut de l'appareil locomoteur et de l'arthrite
Institut des neurosciences, de la santé mentale et des toxicomanies
Institut de la nutrition, du métabolisme et du diabète
Institut de la santé publique et des populations

Le 28 février 2022

Docteur Annie Leblanc
University Laval
Faculty of Medicine
1050 Avenue de la Médecine
Pavillon Ferdinand-Vandry, Local 4617
Quebec city, Québec G1V 0A6

Docteur Leblanc,

Au nom des Instituts de recherche en santé du Canada (IRSC) nous avons le plaisir de vous informer que la demande de subvention que vous avez présentée récemment au concours Subvention Catalyseur : Quatre objectifs et équité qui s'intitule "Clinical integration of digital patient-reported outcomes in primary healthcare for chronic diseases management - a systematic review (inPR1nt)" a été approuvée. Les documents relatifs à vos évaluations se trouvent sur RechercheNet. Veuillez noter que votre autorisation de financement suivra par courrier.

Étant donné que les IRSC n'avisent pas les co-candidats, nous vous prions de communiquer le résultat de cette demande aux personnes concernées ainsi qu'à leur établissement de recherche (s'il diffère du vôtre).

Veuillez noter que la section des conditions de financement de la possibilité de financement mentionne que :
(1) « Les titulaires de subvention doivent participer à une réunion virtuelle de mi-parcours afin de communiquer les résultats qui se dégagent de leurs travaux et les premières leçons apprises. Les IRSC organiseront cette rencontre. » Les IRSC ont décidé de remplacer la réunion virtuelle de mi-parcours par une réunion de fin de subvention. Les titulaires de subvention doivent participer à la réunion de fin de subvention, qui servira de forum pour partager les résultats finaux, les implications politiques et les leçons apprises. Le responsable stratégique, l'Institut des services et des politiques de la santé des IRSC, communiquera avec vous pour plus de détails.
(2) En plus du rapport final, les titulaires de subvention doivent remettre une synthèse en bref de deux pages résumant leurs constatations et faire ressortir les considérations stratégiques et/ou les solutions applicables au contexte canadien (c.-à-d. les options les plus prometteuses pour les administrations au Canada sur le plan de l'amélioration significative des résultats). Un modèle sera fourni d'avance aux équipes. Le modèle et les instructions vous serons transmis sous peu.

Pour toute question veuillez communiquer avec l'équipe de soutien des IRSC par courriel à support-soutien@cihr-irsc.gc.ca. Nous vous prions de ne pas communiquer avec les agents ou les membres du comité d'examen par les pairs.

Vous félicitant de votre succès à ce concours, nous vous prions d'agréer nos salutations distinguées.

Sincèrement,

AJ Jackson

Aaron Jackson
Acting Manager, Program Design and Delivery
Research Programs Portfolio

Canada

CIHR IRSC
Discoveries for life / Découvertes pour la vie

Canadian Institutes of Health Research
160 Elgin Street, 9th Floor
Address Locator 4809A
Ottawa, Ontario K1A 0W9

Instituts de recherche en santé du Canada
160, rue Elgin, 9e étage
Indice de l'adresse 4809A
Ottawa (Ontario) K1A 0W9

Institute of Aging
Institute of Cancer Research
Institute of Circulatory and Respiratory Health
Institute of Gender and Health
Institute of Genetics
Institute of Health Services and Policy Research
Institute of Human Development and Child and Youth Health
Institute of Indigenous Peoples' Health
Institute of Infection and Immunity
Institute of Musculoskeletal Health and Arthritis
Institute of Neurosciences, Mental Health and Addiction
Institute of Nutrition, Metabolism and Diabetes
Institute of Population and Public Health

Institut du vieillissement
Institut du cancer
Institut de la santé circulatoire et respiratoire
Institut de la santé des femmes et des hommes
Institut de génétique
Institut des services et des politiques de la santé
Institut du développement et de la santé des enfants et des adolescents
Institut de la santé des Autochtones
Institut des maladies infectieuses et immunitaires
Institut de l'appareil locomoteur et de l'arthrite
Institut des neurosciences, de la santé mentale et des toxicomanies
Institut de la nutrition, du métabolisme et du diabète
Institut de la santé publique et des populations

Le 28 février 2022

Docteur Annie Leblanc
University Laval
Faculty of Medicine
1050 Avenue de la Médecine
Pavillon Ferdinand-Vandry, Local 4617
Quebec city, Québec G1V 0A6

Bonjour,

Au nom des Instituts de recherche en santé du Canada (IRSC), j'aimerais vous féliciter de votre succès au récent concours de financement des IRSC!

En tant que chercheurs du domaine de la santé, nous partageons un objectif commun : améliorer la santé et le bien-être des populations, au Canada et dans le monde. Par l'entremise des IRSC, le gouvernement du Canada offre un soutien essentiel aux chercheurs canadiens œuvrant dans tous les thèmes de la recherche en santé, qui sont étroitement liés, afin d'améliorer la santé de toute la population canadienne. Titulaire d'une subvention des IRSC, vous faites maintenant partie de cette entreprise.

Vous n'êtes pas sans savoir que l'évaluation de votre demande de subvention a été possible grâce aux pairs évaluateurs qui ont généreusement donné de leur temps pour soutenir le secteur canadien de la recherche en santé. En tant que titulaire de fonds des IRSC, vos connaissances et votre expertise sont fort précieuses. C'est pourquoi je vous encourage à participer à l'évaluation par les pairs, lorsqu'une invitation à cet effet vous sera transmise, et à envisager de devenir membre du Collège des évaluateurs, si vous ne l'êtes pas déjà. Veuillez consulter la page www.cihr-irsc.gc.ca/f/49923.html pour prendre connaissance des critères de sélection et savoir comment soumettre votre candidature.

Aujourd'hui, l'activité scientifique est surveillée de près et il est plus important que jamais de veiller à ce que notre travail soit bien compris. Je vous demande donc de faire mention du financement des IRSC dans vos présentations et vos communications au sujet de vos recherches, et de continuer de faire valoir l'importance cruciale de la recherche pour la santé des Canadiennes et des Canadiens. Vous trouverez à l'adresse https://cihr-irsc.gc.ca/f/30789.html des moyens de reconnaître le soutien reçu et découvrirez comment l'équipe des Communications des IRSC peut vous aider à faire connaître votre recherche.

Encore une fois, je tiens à vous féliciter de cet exploit et à vous offrir mes meilleurs vœux de succès dans toutes vos entreprises. Je suivrai avec intérêt l'avancement de vos recherches.

Je vous prie de recevoir mes plus cordiales salutations.

Michael J. Strong, M.D., FRCPC, FAAN, MACSS
Président

503632-202110QAE-QAE-475355-I006-147743-CONGR

CIHR IRSC
Discoveries for life / Découvertes pour la vie

Canada

Review Type/Type d'évaluation: Committee Member 1/Membre de comité 1
Name of Applicant/Nom du chercheur: Leblanc, Annie
Application No./Numéro de demande: 475355
Agency/Agence: CIHR/IRSC
Competition/Concours: 2021-10-26 Catalyst Grant: Quadruple Aim and Equity/Subvention Catalyseur : Quatre objectifs et équité
Committee/Comité: Catalyst Grant : Quadruple Aim and Equity/Subvention catalyseur : Quatre objectifs et équité
Title/Titre: Clinical integration of digital patient-reported outcomes in primary healthcare for chronic diseases management - a systematic review (inPR1nt)

Assessment/Évaluation:

Scientific Merit:

1. Concept: Quality of the Idea.

Overall goals and objectives are well-defined and clearly linked to the objective of advancing and informing progress towards quadruple aim and equity for all, focused on implementation of PROMS in primary care to advance chronic disease management. The project is solely focused on a detailed and robust systematic review of evidence. It does not reach further to define recommendations or strategies to introduce PROMS for primary care, although this is implied. The project clearly responds to the objectives of advancing Quadruple Aim by documenting and defining outcomes measurement in primary care for management of chronic illness. Makes a strong case for the importance of outcomes measurement in primary care to document achievement of quadruple aim.

2. Feasibility:

Approaches, methods, and integration of sex and gender not clearly described relative to proposed outputs or contributions. Sex and gender are briefly acknowledged, no mention of particular analysis of how current evidence informs or documents sex and gender considerations.Applicant team and knowledge users described well, clearly engaging knowledge users in the iKT process. Timelines and deliverables are feasible.

Team's expertise and experience
PI has an extensive track record of funding and publications relevant to primary care, patient oriented research, less focus on quadruple aim in health systems. Engagement of the applicant team described briefly, but no specific tasks of each is described. Potential research foundation for collaborative research not clearly defined, but could have been.
iKT plan is strong, well developed, accessing national networks of leaders and collaborators via SPOR networks across Canada.

Potential Impact.
The project addresses high-priority evidence needs relative to the integration of PROMS into primary care to advance patient outcomes, workforce outcomes, quality, safety and cost. Relies on a systematic review of literature, stops short of advancing findings in the context of Canadian primary care, which would have further strengthened the potential impact.
Project will inform future decisions, policies, or actions that advance achievement of the Quadruple aim,

**Review Type/Type d'évaluation:**	Committee Member 1/Membre de comité 1
**Name of Applicant/Nom du chercheur:**	Leblanc, Annie
**Application No./Numéro de demande:**	475355
**Agency/Agence:**	CIHR/IRSC
**Competition/Concours:**	2021-10-26 Catalyst Grant: Quadruple Aim and Equity/Subvention Catalyseur : Quatre objectifs et équité
**Committee/Comité:**	Catalyst Grant : Quadruple Aim and Equity/Subvention catalyseur : Quatre objectifs et équité
**Title/Titre:**	Clinical integration of digital patient-reported outcomes in primary healthcare for chronic diseases management - a systematic review (inPR1nt)

Assessment/Évaluation:

however offers no actual evidence of implementation of PROMS in the Canadian context, and does not identify plan to create recommendations for PROMS introduction into primary care in Canada. Team's iKT plan is supported by a strong track record of leadership and engagement of health leaders, sector leaders in Canada.

Review Type/Type d'évaluation: Committee Member 2/Membre de comité 2
Name of Applicant/Nom du chercheur: Leblanc, Annie
Application No./Numéro de demande: 475355
Agency/Agence: CIHR/IRSC
Competition/Concours: 2021-10-26 Catalyst Grant: Quadruple Aim and Equity/Subvention Catalyseur : Quatre objectifs et équité
Committee/Comité: Catalyst Grant : Quadruple Aim and Equity/Subvention catalyseur : Quatre objectifs et équité
Title/Titre: Clinical integration of digital patient-reported outcomes in primary healthcare for chronic diseases management - a systematic review (inPR1nt)

Assessment/Évaluation:

Applicants propose to conduct a systematic review of the literature on the impact of digital PROMs in primary healthcare and to identify effective implementation strategies. The review of the literature is incomplete with previews reviews missing. In addition, work published by CIHI and by APERSU on PROMs is not included. The application fails to provide convincing information on the need for this research or on future research that would build on this project.

1: Concept
Quality of the idea:
o The overall goals and objectives of the project are not well-defined and clear, with distinct outputs and anticipated outcomes that will support advances in knowledge, research, health care, health systems, and/or health outcomes related to one or more of the Quadruple Aim goals and health equity for all.

2: Feasibility
Approach
o The approaches, methods and integration of sex and gender appropriate to deliver the proposed output(s) and achieve the proposed contribution(s) are feasible.
Team’s expertise and experience
o The applicant team does not appear to be aware of Canadian initiatives.

3: Potential Impact
The application does not state to what extent the project will inform future decisions, policies or actions that advance achievement of the Quadruple Aim goals and health equity for all?

Review Type/Type d'évaluation: SO Notes /Notes de l'agent scientifique
Name of Applicant/Nom du chercheur: Leblanc, Annie
Application No./Numéro de demande: 475355
Agency/Agence: CIHR/IRSC
Competition/Concours: 2021-10-26 Catalyst Grant: Quadruple Aim and Equity/Subvention Catalyseur : Quatre objectifs et équité
Committee/Comité: Catalyst Grant : Quadruple Aim and Equity/Subvention catalyseur : Quatre objectifs et équité
Title/Titre: Clinical integration of digital patient-reported outcomes in primary healthcare for chronic diseases management - a systematic review (inPR1nt)

Assessment/Évaluation:

Strengths:

- The committee felt that the area of study (how to incorporate the use of digital PROMs in primary care) was very worthwhile. Primary care and management of chronic disease is a priority for all health systems, and a tool that could effectively engage patients to report their outcomes, particularly if it could be linked to improving self-management was seen as important

- The study methods were felt to be strong, incorporating use of Cochrane methods for systematic review and RE-AIM

- This was felt to be a strong team with very good expertise, including a strong PI who was connected to researchers across Canada

Weaknesses:

- The knowledge translation plan was not as strong as other aspects of this grant. While appropriate knowledge users were involved, to maximize the impact of this work, engagement of additional knowledge users was recommended. For instance, the applicants were encouraged to engage CIHI, the Canadian Network for Digital Health Evaluation, and individuals and knowledge users working in this area in other provinces. The committee felt that other knowledge users would be important to scaling this type of innovation.

Budget:

- No concerns noted

**Review Type/Type d'évaluation:**	SO Notes /Notes de l'agent scientifique
**Name of Applicant/Nom du chercheur:**	Leblanc, Annie
**Application No./Numéro de demande:**	475355
**Agency/Agence:**	CIHR/IRSC
**Competition/Concours:**	2021-10-26 Catalyst Grant: Quadruple Aim and Equity/Subvention Catalyseur : Quatre objectifs et équité
**Committee/Comité:**	Catalyst Grant : Quadruple Aim and Equity/Subvention catalyseur : Quatre objectifs et équité
**Title/Titre:**	Clinical integration of digital patient-reported outcomes in primary healthcare for chronic diseases management - a systematic review (inPR1nt)

Assessment/Évaluation:

****

Note: The final rating of the application, provided in the Notice of Decision (NOD), is the averaged rating of the peer review committee members following the discussion of the application during the committee meeting, and therefore may differ from the ratings provided by the assigned reviewers in their respective reviews.

Remarque : La cote définitive de la demande, qui apparaît dans l'avis de décision, représente la moyenne des cotes accordées par les membres du comité d'évaluation par les pairs après avoir débattu de la demande à la réunion du comité. Elle peut donc différer de celle donnée par les évaluateurs dans leur évaluation respective.
